# Factors associated with advance directives completion among patients with advance care planning communication in Taipei, Taiwan

**DOI:** 10.1371/journal.pone.0197552

**Published:** 2018-07-06

**Authors:** Dachen Chu, Yung-Feng Yen, Hsiao-Yun Hu, Yun-Ju Lai, Wen-Jung Sun, Ming-Chung Ko, Li-Ying Huang, Chu-Chieh Chen, J. Randall Curtis, Ya-Ling Lee, Sheng-Jean Huang

**Affiliations:** 1 Department of Neurosurgery, Taipei City Hospital, Taipei, Taiwan; 2 Institute of Public Health and Community Medicine Research Center, National Yang-Ming University, Taipei, Taiwan; 3 School of Medicine, National Yang-Ming University, Taipei, Taiwan; 4 Department of Health Care Management, National Taipei University of Nursing and Health Sciences, Taipei, Taiwan; 5 Section of Infectious Diseases, Taipei City Hospital, Taipei, Taiwan; 6 Department of Education and Research, Taipei City Hospital, Taipei, Taiwan; 7 Division of Endocrinology and Metabolism, Department of Internal Medicine, Puli Branch of Taichung Veterans General Hospital, Nantou, Taiwan; 8 Department of Exercise Health Science, National Taiwan University of Sport, Taichung, Taiwan; 9 Community Medicine Department & Family Medicine Division, Taipei City Hospital Zhongxing Branch, Taipei, Taiwan; 10 Department of Urology, Taipei City Hospital, Taipei, Taiwan; 11 School of Medicine, College of Medicine, Fu Jen Catholic University, New Taipei, Taiwan; 12 Division of Endocrinology and Metabolism, Department of Internal Medicine, Fu Jen Catholic University Hospital, New Taipei City, Taiwan; 13 Division of Pulmonary and Critical Care Medicine, Harborview Medical Center, University of Washington, Seattle, Washington; 14 Department of Dentistry, Taipei City Hospital, Taipei, Taiwan; 15 Department of Dentistry, School of Dentistry, National Yang-Ming University, Taipei, Taiwan; 16 Department of Surgery, Medical College, National Taiwan University Hospital, Taipei, Taiwan; Duke-NUS Medical School, SINGAPORE

## Abstract

**Background:**

Although advance directives (AD) have been implemented for years in western countries, the concept of AD is not promoted extensively in eastern countries. In this study we evaluate a program to systematically conduct advance care planning (ACP) communication for hospitalized patients in Taiwan and identify the factors associated with AD completion.

**Methods:**

In this retrospective evaluation of a clinical ACP program, we identified adult patients with chronic life-limiting illness admitted to Taipei City Hospital between April 2015 and January 2016. Trained healthcare providers held an ACP meeting to discuss patients’ preference regarding end-of-life care and AD completion. A multiple logistic regression was performed to determine the factors associated with the AD completion.

**Results:**

A total of 2878 patients were determined to be eligible for ACP during the study, among which 1798 (62.5%) completed ACP and data was available for 1411 patients (49.1%). Of the 1411 patients who received ACP communication with complete data, the rate of AD completion was 82.6%. The overall mean (SD) age was 78.2 (14.4) years. Adjusting for other variables, AD completion was associated with patients aged ≥ 85 years [adjusted odds ratio (AOR) = 1.80, 95% CI 1.21–2.67], critical illness (AOR = 1.17, 95% CI 1.06–1.30), and social workers participating in ACP meetings (AOR = 1.74, 95% CI 1.24–2.45).

**Conclusion:**

The majority of inpatients with chronic life-limiting illness had ACP communication as part of this ACP program and over 80% completed an AD. Our study demonstrates the feasibility of implementing ACP discussion in East Asia and suggests that social workers may be an important component of ACP communication with patients.

## Introduction

Advance directives (AD) document patients' wishes with respect to end-of-life (EOL) treatment. First sanctioned in the United States in 1976, AD were designed to protect patient autonomy under the belief that patients who lose decision-making capacity are more likely to receive the care they want if they document their wishes in advance [1]. Prior studies have suggested that patients are more likely to receive care consistent with their goals if they complete AD [2]. Studies have also shown that patients with AD completion are more likely to receive hospice care [2] and die at home [3].

Advance care planning (ACP) is a process that includes, among other components, discussing and completing AD with respect to EOL treatment for patients who may lose decision-making capacity [4]. ACP discussion and AD completion are important because many patients lose their capacity to make decisions regarding the use or avoidance of life-sustaining treatments before they die [2]. A randomized trial from Australia found that ACP and the completion of AD, when implemented on hospitalized patients over the age of 80 years, was associated with decreased ICU use at the end of life, decreased depression and post-traumatic stress symptoms among family members, and increased family satisfaction with care and the patient’s death [5].

In 1992, the US Congress had passed the Patient Self-Determination Act mandating that all Medicare-certified institutions need to provide written information regarding patients' right to formulate AD [2]. Although AD have been implemented for years in western countries, AD is not promoted extensively in Asian countries. Two prior reports from China showed that 96% of the nursing home residents [6] and 81% of the elderly inpatients with chronic diseases [7] had not previously heard of the concept of “ADs”.

In Taiwan, patients’ rights regarding EOL care have been enforced by a recent law. In 2015, Taiwan launched the first Patient Self-Determination Act (PSDA) in Asia. According to PSDA, adults who have full capacity to make juridical decisions can establish AD regarding the EOL care. Hospital institutions are also encouraged to provide written information regarding patients' right to formulate AD. Although patient autonomy regarding the EOL care has been reinforced by this law, there have been no prior studies examining the implementation of ACP in Taiwan.

Several studies have attempted to identify the factors associated with AD completion. Previous reports have found that the factors associated with AD completion included older age [6, 8–12], female gender [10, 12], higher income [9], and non-white race [13]. However, most of these studies were conducted in western countries [8–13]. Factors associated with AD completion may vary in different cultures. Understanding the factors associated with AD completion may help provide opportunities to increase their use.

We sought to implement a systematic ACP program at a large city hospital in Taipei and identify the factors associated with AD completion during ACP among hospitalized patients with chronic life-limiting illnesses. Our goals were to demonstrate the feasibility of a large scale ACP and to identify the factors associated with the completion of AD to help target future ACP programs.

## Methods

### Setting and study population

Taipei City Hospital (TCH) implemented a program to proactively promote ACP for hospitalized patients since 2015. Eligible patients were those who had a) chronic life-limiting illness (e.g., cancer, chronic renal disease, cerebrovascular accident), or b) functional impairment limiting their ability to complete activities of daily living (e.g., bed-ridden).

If the patients agreed, a healthcare provider trained in ACP held an ACP meeting to discuss patients’ treatment and preferences with respect to the EOL care. During the ACP meeting, healthcare providers discussed the following issues with patients and their family: (1) informing patients and family about diagnosis and prognosis; (2) discussion of the goals of medical treatment; (3) discussion of the options for life-sustaining treatment during EOL care. Participants in ACP included patients and their family. Physicians and nurses were required to attend in the ACP meeting. Social workers were invited to participate in the ACP communication with patients if they were available.

Our study included adult patients who were aged ≥ 18 years and completed an ACP meeting with healthcare providers between April 2015 and January 2016. The data for this study were obtained retrospectively from patients' medical records. This study was approved by the Institutional Review Board of TCH (TCHIRB-10502121-E), which waived the requirement for consent.

### Training program for healthcare providers in ACP

To promote ACP and AD among admitted patients, TCH held a series of hospice and palliative care training programs for all healthcare providers in 2015. The hospice and palliative training programs included: (1) education of hospice and palliative care for patients with terminal illness; (2) education of emotional support for the patients and family; (3) training in communication skills with patients during the ACP discussion; (4) education on patients’ rights regarding AD. The training program consisted of thirty-nine hours of didactic training and four hours of simulation training on ACP discussions. All healthcare providers were asked to receive the hospice and palliative training before holding the ACP discussion with the patients.

### Definition of AD completion

In this study, AD completion was defined as a patient or designated health care proxy (e.g., patients’ family) signing an AD during the ACP meeting [14]. The AD completion can be revoked by patients or family at any time.

### Data collection

At the time of ACP communication, patients’ sociodemographic information was collected. The sociodemographic characteristics included age and gender. Critical illness was defined as patients with unstable vital signs such as septic shock or respiratory failure [15, 16]. The comorbidities were determined according to the International Classification of Diseases, 9^th^ Revision, Clinical Modification (ICD-9-CM) and ICD-10-CM code. The comorbidities in the patients included cancer, cerebrovascular accident, congestive heart failure, chronic kidney disease, and chronic obstructive pulmonary diseases. A person was considered to have comorbidity only if the condition was documented in the medical record during the inpatient stay.

### Statistical analysis

Variables associated with AD completion were analyzed using frequencies and percentages for categorical variables, and means for continuous measures. In univariate analysis, Chi-square test was used to evaluate the factors (e.g., age, gender, and comorbidities) associated with the completion of AD. All variables found to have an association, with *p* < 0.10, through the univariate analysis were considered for inclusion in the multivariate analysis. Backward stepwise regression was performed to obtain the final model that included the factors with *P* < 0.05. Odds ratios (OR) and adjusted odds ratios (AOR) with 95% confidence intervals (CI) were reported to show the strength and direction of these associations.

Analyses were conducted using SPSS version 22.0 statistical software packages (SPSS, Chicago IL, USA).

## Results

### Patient and healthcare provider characteristics

A total of 2878 patients were eligible for ACP during the study period, among which 1798 (62.5%) subjects completed the ACP communication. After excluding those with unknown age (n = 1), those with unknown diagnosis (n = 247), and those with unknown ACP participants from patients and their family (n = 139), the remaining 1411 patients were included in the analysis. The overall mean (SD) age of the participants was 78.2 (14.4) years and 56.2% of the participants were male (Table 1). Of 1411 patients, 225 (16.0) subjects were admitted due to malignancy, 195 (13.8) due to pneumonia, 71 (5.0) due to CVA, 53 (3.8) due to chronic renal failure, and 27 (1.9) due to chronic obstructive pulmonary disease.

**Table 1:** Characteristics of patients with advance care planning communication.

Variables	N = 1411, n (%)
**Age (years)**	
Mean ± SD	78.2 ± 14.4
18–64	246 (17.4)
65–74	207 (14.7)
75–84	390 (27.6)
≥85	568 (40.3)
**Gender**	
Female	618 (43.8)
Male	793 (56.2)
**Education level**	
Elementary school or less	491 (34.8)
High school	234 (16.6)
University	514 (36.4)
Unknown	172 (12.2)
**Critical illness**	
No	925 (65.6)
Yes	486 (34.4)
**Comorbidity**	
Malignancy	343 (24.3)
CVA	113 (8.0)
CHF	39 (2.8)
CKD	178 (12.6)
COPD	144 (10.2)
**ACP participants from the healthcare providers**	
Attending physicians	1359 (96.3)
Palliative physician	73 (5.2)
Residents	193 (13.7)
Nursing head	997 (70.7)
Nurses	1105 (78.3)
Social workers	400 (28.3)
**Completion of advance directives**	
No	246 (17.4)
Yes	1165 (82.6)

SD, standard deviation; CVA, cerebral vascular accident; CHF, congestive heart failure; CKD, chronic kidney disease; COPD, chronic obstructive pulmonary disease; ACP, advanced care planning.

Healthcare providers included attending physicians (n = 1359), palliative care physicians (n = 73), residents in internal medicine (n = 193), nurses (n = 2102), and social workers (n = 400). All ACP meetings (n = 1411) included at least one physician; of those, 55 (3.9%) meetings included more than one physician. Nurses were present for 1363 (96.6%) meetings and social workers were present for 400 (28.3%) ACP meetings. Overall, 82.6% of the patients had AD completion during the ACP meeting.

### Univariate analysis for the factors associated with AD completion

Table 2 shows the results of the univariate analysis for the factors associated with AD completion. The chi-square test revealed the variables that were significantly associated with AD completion, including age ≥ 85 years, critical illness, and social workers participating in the ACP meetings.

**Table 2:** Univariate analysis of factors associated with advance directives completion among patients with advance care planning communication.

Variables	Number of patients	AD completion	Univariate	
		n (%)	OR (95%CI)	p value
**Age (years)**				
18–64	246	194 (78.9)	1	
65–74	207	167 (80.7)	1.12 (0.71–1.77)	0.633
75–84	390	311 (79.7)	1.06 (0.71–1.56)	0.789
≥85	568	493 (86.8)	1.76 (1.19–2.60)	0.004
**Gender**				
Female	618	514 (83.2)	1	0.596
Male	793	651 (82.1)	0.93 (0.70–1.23)	
**Education level**				
Elementary school or less	491	413 (84.1)	1	
High school	234	195 (83.3)	0.94 (0.62–1.44)	0.789
University	514	416 (80.9)	0.80 (0.58–1.11)	0.185
Unknown	172	141 (82.0)	0.86 (0.54–1.36)	0.515
**Critical illness**				
No	925	748 (80.9)	1	0.020
Yes	486	417 (85.8)	1.43 (1.06–1.94)	
**Malignancy**				
No	1068	885 (82.9)	1	0.601
Yes	343	280 (81.6)	0.91 (0.67–1.26)	
**CVA**				
No	1298	1074 (82.7)	1	0.552
Yes	113	91 (80.5)	0.86 (0.53–1.41)	
**CHF**				
No	1372	1133 (82.6)	1	0.932
Yes	39	32 (82.1)	0.96 (0.42–2.21)	
**CKD**				
No	1233	1012 (82.1)	1	0.202
Yes	178	153 (86.0)	1.34 (0.86–2.09)	
**COPD**				
No	1267	1044 (82.4)	1	0.626
Yes	144	121 (84.0)	1.12 (0.70–1.80)	
**ACP participants from the healthcare providers**				
Attending physicians	1359	1122 (82.6)	0.99 (0.48–2.06)	0.980
Palliative physician	73	64 (87.7)	1.53 (0.76–3.12)	0.238
Residents	193	167 (86.5)	1.42 (0.91–2.20)	0.118
Nursing head	997	826 (82.8)	1.07 (0.79–1.44)	0.664
Nurses	1105	913 (82.6)	1.02 (0.73–1.42)	0.912
Social workers	400	349 (87.3)	1.64 (1.17–2.28)	0.004

AD, advance directives; OR, odds ratio; CI, confident interval; CVA, cerebral vascular accident; CHF, congestive heart failure; CKD, chronic kidney disease; COPD, chronic obstructive pulmonary disease; ACP, advanced care planning.

### Multivariate Analysis for the factors associated with AD completion

Factors associated with the completion of ADs in the univariate analysis (*p<*0.10) were considered for inclusion in the multivariate analysis. Controlling for all other variables in the model, AD completion was associated with age ≥ 85 years (AOR = 1.80, 95% CI 1.21–2.67), critical illness (AOR = 1.17, 95% CI 1.06–1.30), and social workers participating in ACP meetings (AOR = 1.74, 95% CI 1.24–2.45) (Table 3).

**Table 3:** Multivariate analysis of factors associated with advance directives completion among patients with advance care planning communication.

Variables	AOR (95%CI)	p value
**Age (years)**		
18–64	1	
65–74	1.13 (0.71–1.80)	0.607
75–84	1.09 (0.74–1.63)	0.659
≥85	1.80 (1.21–2.67)	0.003
**Critical illness**	1.17 (1.06–1.30)	0.003
**Social workers participating in ACP**	1.74 (1.24–2.45)	0.001

AOR, adjusted odds ratio; CI, confident interval; ACP, advanced care planning.

## Discussion

In this study of 1411 adult patients with ACP communication, the overall rate of AD completion was 82.6%. We demonstrated the feasibility of a large scale program to train healthcare providers to conduct ACP and found that the majority of these ACP meetings resulted in completion of an AD. Furthermore, after adjusting for all other variables, increased AD completion was associated with patient age ≥ 85 years, having critical illness, and social worker participation in ACP.

Compared to the previous reports, the rate of AD completion in the present study was higher than 73.4% seen among inpatients with congestive heart disease in the US [17]. Furthermore, the rate of AD completion in this study was higher than 22% of AD completion among Hawaii residents [18], and 28% in Asian nursing home residents [19]. Our higher proportion is most likely a consequence of the implementation of an ACP program that included training for healthcare providers. All healthcare providers were encouraged to participate in a palliative care training program which included simulation training before conducting an ACP meeting with the patients. Previous studies have shown that simulation training positively influenced ACP content, including the exploration of patient values/goals, making a recommendation regarding AD, and framing AD within the context of shared decision-making [20, 21]. Our study suggests that a comprehensive palliative care training program for healthcare providers can help promote ACP discussions and completion of an AD.

There is relatively little research on ACP and AD in East Asia and studies suggest that both ACP and AD are less common in this setting [6, 7]. Taiwan has been a leader in Asia with regards to palliative care [22] and had launched the Patient Self-Determination Act in 2015 [23]. All Taiwanese adults who have full capacity to make juridical decisions can establish AD regarding the EOL care and family members can become surrogate decision-makers if patients lose their decisional capacity. The findings of this study suggest that ACP and AD are feasible in the East Asia.

This study found that patients were more likely to have AD completion if social workers participated in the ACP meeting. When healthcare provider held an ACP meeting with patients at TCH, social workers were invited to participant in the ACP meeting if they were available. During ACP meeting, physicians discussed the issue of EOL care with patients. Social workers often offered patients more information regarding their rights of AD during the ACP meeting. Social workers also identified whom among the family were the major medical decision makers. When patients and family felt sad during the discussion of EOL care, social workers offered emotional support. Previous studies reported that social workers have good communication skills and are also trained explicitly to provide spiritual support to the patients [24, 25]. Although this is an observational study, our findings suggests that social workers may plan an important role for the ACP communication with patients.

Consistent with the previous reports, we also found that patient age ≥ 85 years was associated with AD completion [6, 8–12]. Older patients have more chronic comorbidities and are more likely to have had a prior discussion with their healthcare providers regarding the preferences of EOL care [26].

Our study has several limitations. First, this was an observational study and the associations cannot be considered causal. Second, there may be important factors (e.g., income status) associated with AD completion, which were not collected in this study. Third, we don’t have data on the patients who did not have ACP communication and cannot address the reasons this communication did not occur. Future studies are needed to explore these reasons. Finally, the external validity of our findings may be a concern because all our participants were Taiwanese. The generalizability of our results to other areas of Asia or non-Asian ethnic groups thus requires further verification.

## Conclusions

This study found that implementation of palliative care training for healthcare providers and a program to routinely implement ACP for hospitalized patients with chronic life-limiting illness is feasible and successful in Taipei, Taiwan. We found that over 60% of eligible patients had ACP and 82.6% of inpatients who had ACP associated with potential decisions about treatment limitation had AD completion after the ACP discussion. Furthermore, our finding suggesting that social workers may be an important component of ACP communication with patients requires additional study.

## Supporting information

S1 Dataset AD_raw data_2017_12_31. (XLS)
